# Case of Hepatic Abscess—Evacuation of Pus Via Bronchial Tubes—Recovery

**Published:** 1868-12-15

**Authors:** S. T. Odell

**Affiliations:** St. Louis, Miami County, Kansas


CHICAGO MEDICAL JOURNAL.

*Vol. XXV.--DECEMBER* 15, 1868.—*No.* 24.

### CASE OF HEPATIC ABSCESS,—EVACUATION OF PUS, VIA BRONCHIAL TUBES,—RECOVERY.

BY S. T. ODELL, M.D., ST. LOUIS, MIAMI COUNTY, KANSAS.

TUESDAY, September 8, 1868, was called to see Nathan C. Farmer, aged 45, married, of robust build, and massive osseous frame. Patient very thin in flesh, and weak; has an almost constant cough; believes himself ready to die of pulmonary consumption, and really appears to be but the wreck of the strong, muscular man he evidently once was. Obtained from the patient, and his wife, substantially the following statement, as to his condition prior to my seeing him: About the first of June last, being in good health, and (per his own assertion) "about the best man in the county," he was at work on his farm, building fence, when he was attacked with a tolerably severe pain in his right shoulder, which continued, with slight intermission, for near a month. About the same time he first noticed a cough, which troubled him somewhat, especially at night, and which grew worse, in spite of such popular remedies as are generally used by rural persons in cases of "bad colds, coughs," etc. Not being of a disposition to succomb to any slight ailment, he continued, as best he might, his usual farm labors, though there soon supervened to the existing symptoms, a feeling of lassitude and sense of debility; said that when down he could scarcely muster courage to attempt to get up, and that he could only plough a "round" or so, until he was completely tired out, and had to take rest. His appetite was but slightly, if at all, impaired; and he was not conscious of any actual diminution of strength, but rather of endurance. He had no respiratory trouble, save the cough mentioned; no chills; no fever; bowels were regular; no jaundice; and no pain, save in the shoulder. So until about July 1st, 1868, when his cough grew worse, and he was seized with a dull, heavy pain, referred to the right hypochrondriac region. This increased in severity for four or five days, when, in a paroxysm of coughing, he became almost suffocated with a sudden flood of "matter," says he "coughed up nearly a quart before he could quit coughing." For four or five days previous to this expulsion of pus, he had found decubitus on his left side or back, intolerable from the pain it caused, and for the two weeks next preceding the 4th of July, had experienced continually a feeling of drowsiness, and disposition to sleep, which could scarce be resisted, even when in conversation with neighbors who called in to see him.

After this the expectoration of pus was a matter of daily, and almost hourly, occurrence, and it is stated to have been, from the very first, of most horribly fetid odor. At this juncture he first called medical aid, and was treated for *Bronchitis*, grew worse for a month, and changed physicians. The last one employed also treated him for Chronic Bronchitis. I am not aware of the kind of treatment instituted by either of them. He continued growing worse, however, and at the time I was called complained of exhausting night sweats, said he could get no rest of nights, on account of his cough, and was, as above stated, very much emaciated.

On exploration of the chest, I could detect no disease of the lungs, save bronchial inflammation, confined to the lower portion of the right lung,—rather diminished resonance on percussion at that point, and slight interference with the respiratory murmur there. Pulse averaged about 85. I did not, at this time, obtain nearly so complete a history of his case as is given above. He had not told me of the *sudden accession* of stinking pus, to the ordinary sputa which occurred about July 4th, and, although he complained that of mornings the expectoration was more profuse, and smelled worse than during the day. I was disposed to credit this partly to the mere accumulation of mucus in the bronchial tubes, during the hours of recumbency, and partly to the exaggeration of a thoroughly frightened patient. I was led to this conclusion by an examination of the sputa expelled while I was there, which did not reveal the characters he ascribed to it. Although convinced of the gravity of the case, yet as my diagnosis was by no means clear, I confined medication for the present to a mixture of *Muriate of Ammonia*, *Morphia*, and extract of *Taraxacum*, directed to the cough, and tincture of the *Chloride of Iron* as a tonic.

September 18. While conversing with Nathan, he had a paroxysm of coughing, in the course of which he cast up at least three ounces of greenish yellow pus, mixed with mucus, and of the most offensive odor imaginable; nothing ever before assaulted my nostrils to which I can justly compare it. I now saw that I had underrated the value of his previous statements regarding it, and by interrogatories elicited the facts above given. On examination I found no change in the condition of the lungs from that above stated, and so extended my observations to the region of the liver. Found quite a depression externally in the right hypochondriac region, and considerable tenderness on pressure, at a point corresponding to the centre of the line of junction of the epigastric and right hypochondriac regions. The patient now stated that his side "was bulged out for awhile, but when he coughed so hard in July and August it got caved in!" It was with some difficulty that I could detect the inferior margin of the liver under the border of the ribs. Aware that the mere expectoration of pus could not be regarded as diagnostic of Hepatic Abscess, and that an empyema opening into the bronchial tubes, would cause the symptoms recorded; I diligently examined him with reference to the latter affection. I could find no symptom indicating such a condition, save those mentioned, and a slight dullness on percussion over the inferior portion of the right lung. The pneumothorax, which must have been present, had this condition obtained, was absent. There were no signs of any marked compression of the lung. The history of the case did not present any symptoms of the pleuritis, which must have antedated an empyema, and I was led to the conviction that I had an hepatic abscess to deal with; one that had been discharging pus through the lungs for seventy-six days, in undiminished quantities, and with a constant aggravation of the general symptoms. I may, however, except the cough, which had not been so troublesome since the use of the *Ammonia* mixture. I ordered a full nourishing diet, and out door exercise as he could bear it. Gave him *Citrate of Iron* and *Quinia*, gr. v., immediately before each meal, and tincture of *Chloride of Iron*, gtt. xx., two hours after breakfast and dinner, and confined my pulmonary medication to simple anodyne, I also applied a plaster of *Pitch* and *Cantharides* over the right hypochondrium.

September 28. Patient improving. No purulent expectoration, save upon first assuming the erect position on arising from bed in the morning, and then in diminished quantity. Continued treatment internally, removed the plaster, and substituted painting the part with *Tincture of Iodine*, each alternate night.

October 20. C. still improving. Purulent expectoration entirely ceased several days ago. Is gaining flesh and strength rapidly. Discontinued the *Tincture of Iron*. Continued the *Citrate*.

November 12. C. reports himself well; says he quit taking medicine two days ago. He looks rather slim yet, but states that he is able to do a great deal of the work about the farm without feeling wearied. On examination, I find still a marked depression in the right hypochondrium, and percussion reveals the fact that his liver does not occupy nearly the space it should normally.

It is admitted on all hands, that Hepatic Abscess is a rare affection in temperate climates, and the gravity of the affection is also universally conceded. Louis, indeed, is stated to have denied that recovery ever took place in cases of this disease, founding his opinion on the fact that he was never able to discover cicatrices in the liver. Dr. William Stokes, however, mentions meeting a cicatrix in the liver of a person who died in Meath Hospital, Dublin, of chronic enteritis, and who had, while in India some time previously, suffered from Hepatitis. Dr. Stokes, also, makes the statement, that the evacuation of the pus through the diaphragm, and into the lung, is by far the most *favorable* of the internal openings which the abscess may make. Prof. Chapman, (*Thoracic and Abdominal Viscera, Philadelphia*, 1844, *p.* 316), never met with a case in which the pus was avacuated in this manner, admits that such cases have been reported, but speaks doubtingly of all reported recoveries. Gibson (*Surgery*, 7*th* 2*d*, *Vol.* 1, *p.* 137), says few patients recover after the matter has been discharged, via either the lungs or bowels. Watson (*Practice of Physic*) regards this mode of exit for the matter as *fearfully perilous*, and reports having never met with but three such cases. These are all the authorities I have been able to consult on the subject since the occurrence of my case. Excepting Dr. Flint's valuable work on Practice of Medicine, and as the latter is so generally found in the libraries of the profession of to-day, it is hardly worth while to quote therefrom, save the statement that there were, of two hundred and three cases collected by Ronis, one hundred and sixty-two deaths; and of eight cases, uncomplicated with dysentery, and opening through the bronchial tubes, six recoveries.

This statement would not seem to support the assertion of Watson, that such cases were fearfully perilous, or the doubt of Chapman, that they ever recovered. One word as to diagnosis, nothwithstanding the popular idea that pain in the right shoulder invariably points to "disease of the liver," and that my patient complained of pain at that point, I do not think the most veteran routinist would have diagnosticated Hepatic derangement from the symptoms present at the outset of his complaint. Many, indeed most, of the symptoms attending the suppurative process were absent, and nothing certainly pointed to abscess of the liver, prior to the discharge of pus, *per orem*, July 4th. At my first visit (September 8th) the diagnosis was not correctly made out, partly by reason of the patient not giving me a full history of his case, and partly because he did not happen to expectorate any pus in my presence. Once on the right track, the diagnosis was not difficult.

The case is reported partly on account of the rare occurrence of the disease in this climate, and as a slight contribution to the literature of the subject, and partly on account of the interest which attaches to it, from the fact that it was for over two months under routine treatment, and unrecognized; nor can I arrogate any great superiority of skill in diagnosis over those who thus treated the case; for, as the record shows, I made the mistake of treating the patient for ten days, without a just appreciation of "what was the matter," and was at last put in the right road to a diagnosis, by the accident of the patient's expectorating pus, at a moment when I happened to be present. This, when a thorough examination on my part in the first instance could not but have revealed the true state of the case. By experience we are taught; and perhaps it would be better if, in our reports of cases, we more generally than we do, recorded also our mistakes. Such records, however, are, like the disease my patient had, "rare in this climate."

